# Laboratory Milk

**Published:** 1909-01-16

**Authors:** 


### Diseases of Children.

### LABORATORY MILK.

Although the use of laboratory milk in this country has largely fallen into disrepute among those who may be regarded as authorities on the subject of infant feeding, there is still an expectation, in occasional members of the profession and in many anxious mothers, of finding in this milk the one and only food for a particular infant. Hence, it is worth while again to draw the attention of our readers to the advantages claimed for this product and the objections which have been raised in theory and found in practice to be correct.

This mode of feeding was devised and enthusiastically advocated by Rotch in America. It is variously known as the American, the percentage, or the laboratory method of feeding; for it was started in America, based on estimations of the percentage composition of breast-milk and cow's milk, and on the preparation of a corresponding substitute in a milk laboratory. Great care is adopted to obtain a pure milk supply. Healthy cows alone are used, tested with tuberculin to exclude latent tuberculosis, specially fed to ensure as far as possible a milk of almost uniform consistence throughout the year; and special precautions are adopted against contamination during and after the milking process. The milk is conveyed in special ice chests, at or below 45° F., to the milk laboratory and is there separated into cream containing 16 per cent. fat, and separated milk, dirt being also extracted during the process of separation. Further modification has been introduced by the preparation of whey, so as to obtain the whey proteins more easily and in greater amounts in the mixtures. Thus, the materials for the preparation of the milk mixtures consist of cream, separated milk, whey, a 20-per-cent. solution of milk sugar in distilled water, and lime water. These are mixed in such proportions as to provide a mixture of the percentage composition ordered; *e.g.* fat 3, sugar 6, whey protein 0.75, caseinogen 0.5. Such mixtures are generally ordered slightly alkaline, and to be heated for 20 minutes at 167° F.; that is, pasteurised. After the food has been mixed in the required proportions it is put into bottles, each containing sufficient for one feed, stoppered with plugs of non-absorbent wool, heated as ordered, and then cooled at 28° F. and kept in the cooling chamber until sent out.

The advantages claimed are that the milk approximates as closely as possible in composition to breast-milk, that it is sterile, free from dirt, and still maintains a considerable degree of freshness. It is obvious that a mere similarity in percentage composition does not render such modified milk analogous to breast-milk. The fat of cow's milk is not identical with that of human milk; its melting point is higher, its globules are larger. There is evidence that the caseinogen and albumin possess certain differences in different milks. It is difficult to estimate the relative proportions of these two proteins in breast milk and to combine them in like amounts in the laboratory product. Artificial lactose is not necessarily identical with human lactose and is liable to impurities. Furthermore, various organic compounds and ferments are present in fresh milk and are destroyed by heating. The claim, therefore, that the modified milk is identical in composition with the breast-milk cannot be justly maintained. It must also be pointed out that it is easier to separate the fat from the milk by the centrifugal machine, in the form of a rich cream, than it is to recombine it in the milk mixture. It tends to separate out and appear on the surface in the form of droplets of butter.

That such milk is sterile is generally untrue, and were it true it is of doubtful advantage to feed infants on sterilised milk. Pasteurisation, as usually adopted, does not sterilise milk, though it may destroy dangerous organisms. It will not destroy the spores of many such, notably those of the bacillus enteritidis sporogenes. It destroys the harmless lactic acid bacilli and allows the spores of the above-named organism, if present, to develop. The writer has known laboratory milk to undergo decomposition and set up serious digestive disturbance as the result of the growth of the hay bacillus. The milk possesses all the disadvantages of pasteurisation and to a less extent those of sterilisation. It is liable to set up subacute scurvy, debility, anæmia, malnutrition, and delayed development. It does not prevent diarrhœal disorders, and apparently sometimes induces them. The pasteurisation of milk is strongly condemned by many American and English physicians.

The claim that the milk retains a certain degree of freshness is of little value. We have no guarantee. The pasteurisation may prevent souring, the most valuable indication of lack of freshness. Milk treated on these lines may be supplied a week old and yet be apparently as fresh as that just prepared. Stale milk may be used in the process and we have no evidence that it has not been used. Under no conditions can such milk be regarded as fresh.

One of the strongest arguments against the use of laboratory milk is that the constant repetition of feeds of the same composition, amount, and flavour is entirely opposed to nature's method of feeding. The breast-milk varies at each feed; in different women; and even during the same nursing. The baby obtains a first course of milk poor in fat and, if it empties the breast, a last course of almost pure cream. Laboratory milk is both unnecessary and expensive. Those parents who can afford the treatment are those who can provide suitable attendants and appliances for the home modification of milk. Others, for whom a more or less sterile milk supply is temporarily advisable on account of their social position and surroundings, find this milk too expensive.

The home modification of milk is quite simple and sufficient. Ashby recommends that 30 oz. of fresh milk be allowed to stand in a glass bottle in a cool place for four to five hours. The upper third will then contain 10 per cent. fat, the upper half 7.5 per cent. The lower half or two-thirds is replaced by water containing an ounce of milk sugar. The mixtures then contain protein 1.0 to 1.75, fat 3.5 to 3.0, and sugar about 6.0 per cent. Cautley advises that fresh milk be allowed to stand for three hours and the top half separated. Five ounces of this is mixed with 6½ drachms of milk sugar, 1 oz. of lime water, and water 14 oz., making a mixture of protein 1, fat 2, and sugar 5 per cent. It is gradually strengthened by replacing the water by the milk, 1 oz. at a time, until the mixture contains 10 oz. of top milk and the other quantities up to 20 oz., the composition being then protein 2, fat 4, sugar 6 per cent. More fat and less protein can be obtained by using the top third or top fourth of the milk. Simple modifications of the above nature are far superior to laboratory preparations, and their manufacture is within the range of intelligence of most people.

